# A Review on the Involvement of Heat Shock Proteins (Extrinsic Chaperones) in Response to Stress Conditions in Aquatic Organisms

**DOI:** 10.3390/antiox12071444

**Published:** 2023-07-18

**Authors:** Sivakamavalli Jeyachandran, Hethesh Chellapandian, Kiyun Park, Ihn-Sil Kwak

**Affiliations:** 1 Lab in Biotechnology & Biosignal Transduction, Department of Orthodontics, Saveetha Dental College & Hospitals, Saveetha Institute of Medical and Technical Sciences (SIMATS), Saveetha University, Chennai 600077, Tamil Nadu, India; hetheshwaran@gmail.com; 2 Fisheries Science Institute, Chonnam National University, Yeosu 59626, Republic of Korea; kiyunpark@chonnam.ac.kr; 3 Department of Ocean Integrated Science, Chonnam National University, Yeosu 59626, Republic of Korea

**Keywords:** HSPs, oxidative stress, ischemia, crustaceans, fish

## Abstract

Heat shock proteins (HSPs) encompass both extrinsic chaperones and stress proteins. These proteins, with molecular weights ranging from 14 to 120 kDa, are conserved across all living organisms and are expressed in response to stress. The upregulation of specific genes triggers the synthesis of HSPs, facilitated by the interaction between heat shock factors and gene promoter regions. Notably, HSPs function as chaperones or helper molecules in various cellular processes involving lipids and proteins, and their upregulation is not limited to heat-induced stress but also occurs in response to anoxia, acidosis, hypoxia, toxins, ischemia, protein breakdown, and microbial infection. HSPs play a vital role in regulating protein synthesis in cells. They assist in the folding and assembly of other cellular proteins, primarily through HSP families such as HSP70 and HSP90. Additionally, the process of the folding, translocation, and aggregation of proteins is governed by the dynamic partitioning facilitated by HSPs throughout the cell. Beyond their involvement in protein metabolism, HSPs also exert a significant influence on apoptosis, the immune system, and various characteristics of inflammation. The immunity of aquatic organisms, including shrimp, fish, and shellfish, relies heavily on the development of inflammation, as well as non-specific and specific immune responses to viral and bacterial infections. Recent advancements in aquatic research have demonstrated that the HSP levels in populations of fish, shrimp, and shellfish can be increased through non-traumatic means such as water or oral administration of HSP stimulants, exogenous HSPs, and heat induction. These methods have proven useful in reducing physical stress and trauma, while also facilitating sustainable husbandry practices such as vaccination and transportation, thereby offering health benefits. Hence, the present review discusses the importance of HSPs in different tissues in aquatic organisms (fish, shrimp), and their expression levels during pathogen invasion; this gives new insights into the significance of HSPs in invertebrates.

## 1. Introduction

Due to the poikilothermic nature of aquatic animals, minor changes in the environment might lead to stress in fish. Fish are often exposed to various environmental stressors, such as pathogens, toxic gases, trauma, temperature fluctuations, and hypoxia. These factors, often referred to as stressors or stress factors, hold significant importance in determining the sequence of events that unfolds after encountering adverse consequences such as microbial infections, toxic exposure, traumatic injury, radiation, or nutritional deficiencies [1]. According to Selye’s [2] original definition from 1950, a normal metabolism is the objective that an animal strives to maintain or restore in the presence of chemical or physical stimuli. Easton [3] further proposed that stress occurs when an environmental or associated factor pushes an animal’s adaptive responses beyond its standard parameters or severely disrupts the animal’s proper functioning, ultimately reducing the probability of survival. This definition closely aligns with the circumstances observed in aquatic species. The term “general adaptation syndrome” (GAS) is used to describe the changes that occur in response to stress. It encompasses a sequence of biochemical and physiological changes that unfold in three stages: the alarm reaction (stage of resistance), during which adaptations are made to achieve homeostasis under the new conditions; the stage of exhaustion, where adaptations fail to restore homeostasis; and, if homeostasis is not achieved, it leads to a further decline in the probability of survival. The components of GAS are not specific to particular species or stressors, but the overall response to each stressor may vary significantly [4].

Research on the general adaptation syndrome in fish has primarily focused on hormonal and nervous responses. The role of the hypothalamic–pituitary internal axis in the GAS in fish has been extensively reviewed by Sumpter (1997) [5]. The impact of stress-mediated hormonal changes on the immune responsiveness of the animal, leading to increased susceptibility to infection, has been extensively discussed by Wedemeyer (1997) [6]. For further information on this aspect of GAS, readers are referred to these authors. Although the cellular stress response has received less attention in higher animals, fish, and shellfish, it is an important feature of the GAS (Locke, 1997) [7]. Cells typically respond to stress by altering gene expression, resulting in the upregulation of highly conserved proteins, collectively known as heat shock proteins (HSPs). These HSP molecules, produced in response to stressful conditions, not only play a crucial role in the early response to stressors but also contribute to host defenses against neoplasia and chronic pathogens. They may even hold potential as a primary avenue for the development of new vaccines, while being fundamental to evolution and all forms of life. Considering the growing interest in harnessing the induction of HSPs for clinical purposes in human medicine [8], methods for their induction are also emerging for veterinary purposes [9,10]. This review aims to explore the nature of the HSP response, its relevance to aquatic animals and their welfare, and recent research on methods of inducing HSPs in aquaculture, particularly concerning health and welfare issues.

## 2. Different Types of Stress Factors Involved in the Expression of HSPs

### 2.1. Desiccation, Temperature, and Hypoxia/Anoxia Stress

The impact of temperature on organisms is well recognized, as it can influence their physiology [11,12,13], behavior [14], and interactions with other species [15,16]. Thermal fluctuations are considered crucial factors that can disrupt physiological systems at the cellular and molecular levels [17]. Temperature affects molecular and physiological processes, influencing an organism’s activity patterns [18,19]. Aquatic organisms can exhibit physiological responses to acute temperature fluctuations before exhibiting behavioral responses [20]. Studies on marine species have shown that their thermal tolerance limits are determined by the onset of hypoxemia, which triggers the activation of anaerobic metabolic pathways [21]. In rocky shores, temperature and desiccation are recognized as key factors that set the upper limits of species distribution, with extreme desiccation stress leading to the diapause of crustacean eggs. However, organisms have developed adaptive mechanisms, including thermal tolerance, heat shock protein expression, and protein thermal stability, to counteract environmental extremes and minimize cell damage. The cellular stress response is activated to maintain cellular function and enhance the organism’s ability to cope with challenging situations [22]. This response involves the activation of cellular pathways such as proteolysis through the ubiquitin–proteasome pathway and the increased production of heat shock proteins [23].

### 2.2. Osmotic Stress

Osmotic stress is a prevalent environmental factor that affects aquatic organisms. Osmoregulation, which is vital in maintaining osmotic homeostasis, plays a crucial role in response to this type of stress. The influence of stressors such as temperature or salinity on organisms has been studied extensively [24,25]. These stressors can impact the osmoregulation capability of organisms by affecting Na+-K+ ATPase activity or inducing heat shock protein production [26], both of which contribute to maintaining relative osmotic hemolymph homeostasis [27]. Numerous studies have investigated the expression patterns of heat shock proteins (HSPs) under salinity stress. For example, the expression of HSP90 was induced in *Crassostrea hongkongensis* [28] and *Eriocheir sinensis* [26,29] under osmotic stress. High salinity stress led to the significant upregulation of HSP70 expression in the hemocytes of *Scylla paramamosain* [30]. In the hepatopancreas of *Portunus trituberculatus*, HSP60, HSP70, and HSP90 showed either downregulated or upregulated expression profiles when exposed to low salinity (4 ppt) [31]. These findings suggest that HSPs play a role in mediating the effects of salinity stress in aquatic crustaceans.

### 2.3. Ultraviolet Radiation Stress

Ultraviolet (UV) radiation, an abiotic factor, can have detrimental effects on organisms, both directly and indirectly. Direct exposure to UV radiation can lead to changes in protein synthesis and DNA due to the absorption of high-energy photons. Indirectly, UV radiation can generate reactive oxygen species that cause damage to proteins, nucleic acids, and lipids [32,33,34]. The impact of UV radiation on aquatic organisms has become a significant concern in recent years. Research conducted on calanoid copepods has shown that UV-induced stress can impair feeding mechanisms and digestion and disrupt the entire food chain [35]. UV radiation directly and indirectly influences the survival, growth, and reproduction of organisms, and it led to the increased expression of antioxidant enzymes and heat shock protein (HSP) genes in the copepod *Paracyclopina nana* [34].

### 2.4. Heavy Metal Stress

Heavy metals pose a significant problem as a cause of pollution in water, soil, and plants. They enter water sources through seepage from household or industrial waste, resulting in serious risks to aquatic ecosystems and aquaculture animals. In laboratory studies focusing on crustaceans, the impact of heavy metals on gene expression changes has been extensively examined. Commonly tested heavy metals include copper (Cu), silver (Ag), zinc (Zn), lead (Pb), manganese (Mn), arsenic (As), and cadmium (Cd) [36,37,38]. Heavy metal stress is closely linked to the induction of oxidative stress. In seawater, heavy metals can trigger oxidative stress in various organisms, including the marine crab *Portunus trituberculatus* [39]. This type of oxidative stress disrupts the cellular redox balance, prompting a protective stress response. Numerous studies on aquatic organisms, particularly crustaceans, have demonstrated that heavy metal stress significantly stimulates the synthesis of antioxidant enzymes [40] and heat shock proteins [26,38]. Heat shock proteins (HSP) appear to play a crucial role in the innate immune systems and stress responses of crustaceans [36,37,38].

### 2.5. Effect of Endocrine Disruptor Chemicals in Heat Shock Proteins

Endocrine disruptor chemicals (EDCs) are compounds that imitate natural hormones, inhibiting their activity or altering their normal regulatory function within the immune, nervous, and endocrine systems [41]. These chemicals are ecotoxicologically significant as they have a tendency to be absorbed onto humic material or accumulate in aquatic organisms, persisting in water or the food web for extended periods. Consequently, their effects can induce prolonged stress in aquatic organisms. Various EDCs, including pesticides, bisphenol A, phthalates, dioxins, and phytoestrogens, have been shown to interact with the female reproductive system and cause endocrine disruption [42]. Endosulfan and deltamethrin, commonly used pesticides in shrimp farms [43], are particularly noteworthy. Endosulfan is widely employed as a broad-spectrum insecticide, primarily in agriculture, and is highly toxic to aquatic organisms [44,45,46]. Studies investigating the stress response induced by EDCs have indicated the significant induction of heat shock protein (HSP) family proteins [47,48,49], detoxification enzymes such as glutathione S-transferases [50], and superoxide dismutase. These proteins are considered to potentially contribute to the protection of aquatic organisms against stress.

### 2.6. Other Toxicants

Apart from the previously mentioned primary chemicals, there exist a significant number of other toxic substances in the habitats of aquatic organisms. These toxicants include hydrocarbons, diatom toxins, emamectin benzoate, nitrite, and prooxidant chemical hydrogen peroxide (H_2_O_2_), among others. They accumulate in aquatic and/or terrestrial environments through the release of household and/or industrial waste. Research studies have demonstrated that these toxicants can have harmful effects on crustaceans [51,52]. In a study conducted by Lauritano et al. [41], it was observed that feeding on a diatom species (*Skeletonema marinoi*) that produced strong oxylipins for only two days led to the significant downregulation of heat shock proteins (HSP40 and HSP70) in the copepod *Calanus helgolandicus*. Diatom oxylipins are known to induce the generation of free radicals, including reactive oxygen species, which can cause oxidative stress and cellular damage. Furthermore, nitrite is considered one of the most prevalent pollutants in aquaculture due to its numerous integrated effects. A study on shrimp demonstrated that oxidative stress was one of the mechanisms of nitrite toxicity [53]. Guo et al. [53] confirmed that exposure to nitrite induced the expression of apoptosis-related genes in hemocytes, while also upregulating the expression levels of HSP70 and antioxidant enzymes to protect against nitrite-induced stress.

## 3. The Role of Heat Shock Proteins in Aquaculture Disease Management

### 3.1. Immunology and Stress Response

The identification of heat shock proteins (HSP) initially occurred in *Drosophila busckii* as a response to stress [54]. Since then, their roles as chaperones in protecting cellular proteins from denaturation have garnered significant interest [55,56]. In aquaculture animals, HSPs have been the focus of numerous studies due to their crucial function in mitigating the stress-induced denaturation of client proteins, as well as their involvement in protein folding, assembly, degradation, and gene expression regulation [57,58]. Physiological and environmental stressors, including high thermal shock, heavy metals, free radicals, desiccation, and microbial infection, can induce the synthesis of HSPs. This induction is considered a vital protective response that is conserved across organisms, enabling them to adapt to environmental challenges. Recent research has revealed the involvement of heat shock chaperonins in autoimmune and innate immune responses in various species, including crustaceans. HSPs play a crucial role in mounting protective immune responses against bacterial and viral diseases [59,60]. In the crustacean aquaculture industry, which faces substantial economic losses due to environmental stressors, investigations into heat shock proteins have gained popularity. These proteins play vital roles in conferring resistance to diverse stressors. Extensive research has been conducted to understand the structures, functions, cross-talk, immune response mechanisms, and innate immune pathways of HSPs in crustaceans when exposed to various environmental stressors or xenobiotics. Exploiting HSPs as a means of preventing and treating aquaculture diseases in commercially cultured aquatic organisms is crucial as it provides an alternative to the use of antibiotics and therapeutic drugs [61]. Furthermore, previous studies have aimed to identify effective strategies for the management of environmental stressors in aquaculture settings for aquatic organisms [62].

### 3.2. Crustaceans: Exploring the Link between Environmental Stresses and Disease

Crustacean aquaculture plays a significant role in the economies of several countries worldwide. However, the expansion and intensification of aquaculture farms have led to the emergence of various new diseases in commercially cultivated species. Disease outbreaks caused by viruses, bacteria, and environmental stressors pose a serious threat to the global crustacean aquaculture industry, resulting in substantial economic losses. Unlike vertebrates, invertebrates lack true adaptive immunity and have developed defense systems that respond to physiological and environmental stresses [16,63]. During crustacean aquaculture, organisms are constantly exposed to environmental stimuli and a range of natural and anthropogenic stressors (Table 1). Numerous studies have demonstrated that physical stressors such as temperature, salinity, and UV radiation, as well as chemical stressors such as endocrine disruptor chemicals, heavy metals, hydrocarbons, and other toxicants, can be detrimental to crustacean cells. Moreover, in natural ecosystems, multiple environmental forces interact, resulting in situations of combined stress [64,65]. Crustaceans possess an innate immune system, which serves as their first line of defense and responds to natural and anthropogenic stimuli, pollutants, and toxins [41,66]. Studies have indicated that certain metabolic enzymes (such as cytochrome P450, glutathione S-transferase, superoxide dismutase, etc.), heat shock proteins, and immune-related proteins in crustaceans play a role in enhancing disease tolerance and aiding the elimination of harmful compounds from their bodies [41,67].

#### Shellfish Diseases and the Role of Pathogens

Shellfish diseases are prevalent and frequently observed in various commercially exploited crustacean species. Currently, a range of pathogens, including *Vibrio*, chitinoclastic bacteria, *Aeromonas, Spiroplasma*, *Rickettsia*-like organisms, *Chlamydia*-like organisms, *Rhodobacteriales*-like organisms, white spot syndrome virus (WSSV), yellow head virus (YHV), *infectious myonecrosis virus* (IMNV), *Enterocytozoon hepatopenaei* (EHP) microsporidian parasites, and covert mortality *nodavirus* (CMNV), have been identified as causes of disease in crustaceans [93]. *Vibrio* species, found in various marine and freshwater crustaceans, are widespread worldwide. *Vibrio* infections commonly result in bacteremia and shell diseases [94]. For instance, *Vibrio parahaemolyticus* infection caused acute hepatopancreatic necrosis disease (AHPND) and led to significant mortality in a penaeid shrimp aquaculture [95]. Chitinolytic or chitinoclastic bacteria are often associated with shellfish diseases, leading to unsuccessful molting in crustaceans [96] or septicemic infections caused by opportunistic pathogenic bacteria [97]. Infections by other pathogens, such as *Rickettsia*-like organisms, *Chlamydia*-like organisms, *spiroplasma*, and *Rhodobacteriales*-like organisms, have caused severe stress or fatal diseases in crustaceans. Efforts have been made by numerous researchers to find effective methods to control bacterial diseases. Recent studies have shown that synbiotics can induce penaeid shrimp immunity and promote the growth of aquatic animals [98]. Oxytetracycline has been found to be highly effective in treating *spiroplasma* disease [99]. Several immune-related genes and proteins, including tachylectin-like genes and proteins and heat shock proteins [67], have been identified as being involved in shrimp tolerance to AHPND-causing strains. Crustacean fibrinogen-related proteins have also been found to participate in the innate immune response during AHPND or other pathogen infections [100]. Additionally, viruses continue to pose a significant challenge to crustacean aquaculture. Recent research has highlighted several new and emerging diseases in shrimp, including hepatopancreatic microsporidiosis, hepatopancreatic haplosporidiosis, aggregated transformed microvilli, covert mortality disease, white spot disease, yellow head disease, infectious myonecrosis, and white tail disease, which represent major viral threats to commercially cultivated shrimp [93].

### 3.3. Expression of Heat Shock Proteins in Fish

**Figure 1:**
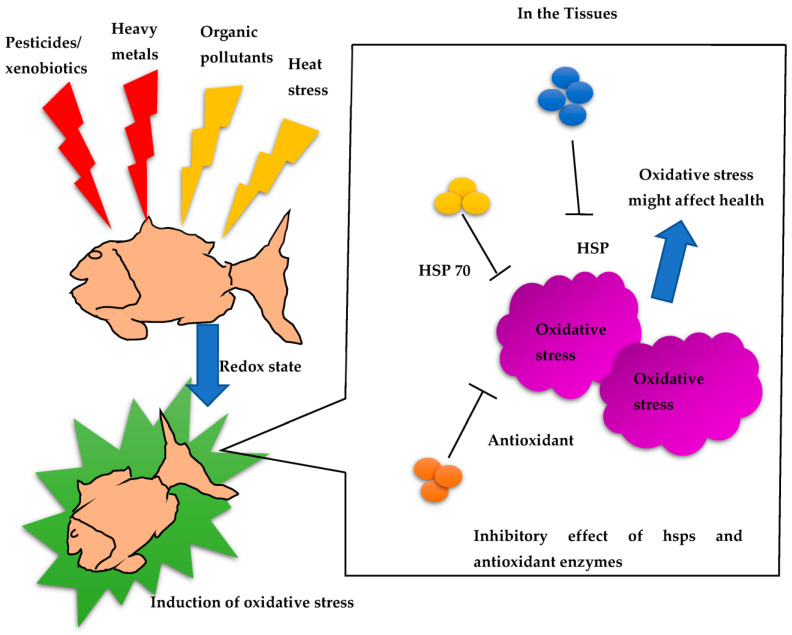
Redox signaling mechanisms and inhibitory effects of HSPs in fish.

The presence of heat shock proteins (HSPs) in fish has been extensively documented, emphasizing their importance in responding to stress and safeguarding cellular integrity. HSPs are a group of highly conserved proteins that serve as molecular chaperones, aiding in the folding, assembly, and breakdown of other proteins. Fish exhibit increased HSP production when exposed to various stressors, such as elevated temperatures, exposure to heavy metals, oxidative stress, and infection by pathogens. Numerous studies have observed the heightened expression of HSPs in different fish species, including zebrafish (*Danio rerio*), rainbow trout (*Oncorhynchus mykiss)*, and gilthead seabream (*Sparus aurata*), in response to stressors [101,102] (Table 2). These HSPs play a vital role in maintaining cellular balance, facilitating fish survival, and enabling adaptation to adverse environmental conditions. Furthermore, HSPs have been implicated in fish immune responses, enhancing their ability to defend against bacterial and viral infections [103]. The monitoring of HSP expression in fish serves as a valuable method to assess environmental stress levels and evaluate the overall health of fish populations in aquatic ecosystems (Figure 1).

### 3.4. Expression of Heat Shock Proteins in Mollusk

**Figure 2:**
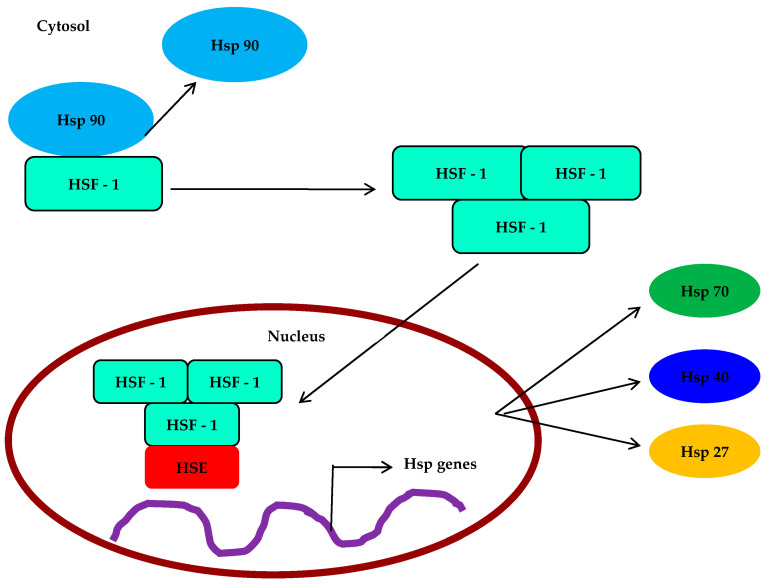
Expression and molecular mechanisms of HSP and HSF in Mollusca.

The expression of heat shock proteins (HSPs) is associated with important developmental processes in various species, including gametogenesis, embryogenesis, and metamorphosis. In marine invertebrates with a biphasic life cycle, where pelagic larvae undergo settlement and metamorphosis, research has revealed interesting findings. For instance, studies on Eastern oyster *C. virginica* larvae and early spat have shown the presence of three HSP70 isoforms: HSC77, HSC72, and HSP69. The expression of constitutive and inducible forms of HSP70 differs among the larval and early juvenile stages and in response to thermal stress. Interestingly, the low expression of HSP69 during early larval and spat development may contribute to their vulnerability to environmental stress. In another investigation, Gunter and Degnan examined how the marine gastropod *Haliotis asinine* expresses HSP90, HSP70, and the heat shock transcription factor (me) during development (Table 3). HSP70, HSP90, and HSF are first expressed in this species by maternal contribution, before being gradually confined to the micromere lineage after cleavage (Figure 2). These proteins are expressed in distinct ways in the prototroch, foot, and mantle during larval morphogenesis. When cells are differentiating and undergoing morphogenesis, their expression is at its highest; however, after morphogenesis is complete, it starts to decline.

### 3.5. Heat Shock Protein Expression in Insects

A group of conserved polypeptides collectively known as heat shock proteins (HSPs) are rapidly increased in synthesis by insects in response to high temperatures and a variety of chemical and physical stimuli. Hspshave molecular-weight-based names, such as Hsp10, Hsp40, Hsp60, Hsp70, Hsp90, and Hsp100. Small Hsps (sHsp) are a subclass of Hsps that play a role in the folding and unfolding of other proteins (Table 4). In the fruit fly *Drosophila busckii*, Ritossa was the first to note that heat and the metabolic uncoupler dinitrophenol caused a distinctive pattern of puffing in the salivary gland chromosomes [54]. This discovery ultimately helped to identify the Hsps that these puffs were representing. The first observation of the increased production of certain proteins in *Drosophila* cells in response to stressors such as heat shock was made in 1974 [170]. There is currently a vast body of research that describes the extensive spectrum of action taken by cells in response to a wide range of biotic and abiotic stressors in a variety of insects [171,172].

### 3.6. Heat Shock Proteins in Myxozoan Parasites (Cnidaria)

Heat shock proteins (HSPs) are expressed by parasites as a response to various stimuli, such as heat and oxidative stress. These HSPs provide parasites with resistance to these harsh conditions, which is crucial for their survival. The genes associated with protein refolding, including HSP60, HSP70, and HSP80 family members, express these heat shock proteins. Apart from their role in protein refolding, HSPs also show significant involvement in other processes, such as maintaining protein balance and stability. They have the ability to bind to abnormal forms of proteins and facilitate their folding into their natural conformations. *T. bryosalmonae*, a parasite, faces the challenge of overcoming the robust immune responses mounted by both brown trout and rainbow trout [185]. This challenge potentially affects various physiological processes of *T. bryosalmonae*, including protein structure and function. Moreover, HSPs found in several parasites, such as *T. cruzi* [186] and Schistosomes [187], have been discovered to elicit an immune response in their respective hosts and are immunogenic in nature. In a recent study, it was found that myxozoan parasites such as *Ceratonova shasta*, *Myxobolus cerebralis*, and *Sphaerospora molnari* from the intestine and abdominal cavity (ascitic fluid) of rainbow trout expressed HSP70 when exposed to oxidative stress [188].

## 4. Defense Mechanisms of Heat Shock Proteins

The heat shock protein family, such as HSP70, are primarily studied for disease control purposes, but other members, such as small heat shock proteins (sHSPs), including HSP60 and HSP90, alongside HSP40/co-chaperone, have shown potential in treating pathogen infections. sHSPs act as oligomeric platforms, binding structurally perturbed proteins without requiring ATP, thereby preventing their irreversible denaturation under cellular stress. HSP90, HSP70, and HSP60 are stress-induced and provide protection against irreversible protein denaturation. However, their primary function involves binding and folding newly synthesized proteins through allosteric rearrangement, which is driven by ATP, although the mechanisms of action and the molecular structure differ among the chaperone families. In cooperation, these HSPs form intracellular networks with accessory proteins and other co-chaperones. sHSP monomers are a group of conserved α-crystallin domains flanked by carboxyl- and amino-terminal sequences and assemble into oligomers. The α-crystallin domain facilitates monomer dimerization and substrate binding, with the efficiency depending on the terminal region. During stress, sHSP oligomers may undergo structural rearrangement or disassemble, which promotes substrate protein interactions and increases surface hydrophobicity. Upon stress resolution, proteins released from sHSPs have the ability to spontaneously refold with the assistance of HSP70, which is an ATP-dependent HSP [189]. The key role of sHSPs is to prevent protein denaturation, which is irreversible during infection and stress.

In aquatic organisms, such as the white shrimp (*L. vannamei*) and *Scrippsiella trochoidea*, various HSP genes (e.g., LvHSP40, LvHSP60, LvHSP70, LvHSC70, and LvHSP90) are significantly induced under acute thermal stress, highlighting their sensitivity to temperature fluctuations. Shrimp HSPs are also highly expressed in response to pathogen infections, as demonstrated by the upregulation of LvHsp60 in the gills, hemocytes, and hepatopancreas after challenge with Gram-negative or Gram-positive bacteria. Furthermore, the use of plant-based polyphenolic compounds such as phloroglucinol and carvacrol has been shown to result in the induction of HSP70 and protection against bacterial infection in brine shrimp and freshwater prawns [190]. These findings suggest that HSPs may play a role in crustaceans’ immune system regulation, which triggers immune defense against diseases, as evidenced by the modulation of immune-related genes. Overall, the investigation of HSPs in aquatic organisms provides insights into their involvement in combating stress and infection, offering potential avenues for disease control and enhancing the immune responses in these organisms.

Biotic stress factor bacteria induce HSP20 expression in fish [191,192]. Similarly, some sHsp cDNA have been isolated and characterized in an expression analysis performed in fish [117,128,193,194,195]. Following this, the HSP expression level was detected in *Ictalurus punctatus* [196], *Paralichthys olivaceus* [197], and *Epinephelus coioides* [198]. However, the expression patterns of fish sHsp under environmental stress are still limited with regard to biotic stress factors. Another type of HSP21 transcript was induced after 24 h exposure to *Vibrio harveyi* in shrimp *P. monodon* [158]; this was found to be entirely different in WSSV infection with *P. monodon* [199]. *M. rosenbergii* showed upregulated expression of HSP37 mRNA in the hepatopancreas under an infectious hypodermal and hematopoietic necrosis virus challenge [200]. In disk abalone *Haliotis discus*, HSP20 expression reached its highest peak in *V. parahemolyticus* with the VHSV virus [201]. Although some sHsp cDNA have been isolated and characterized in fish, there is little research on their roles in the immune response [117,128,193,194,195]. Recently, it was validated that, when infected with Singapore grouper iridovirus (SGIV) and *V. alginolyticus*, *Epinephelus coioides* hsp22 mRNA expression was significantly increased, and HSP22 could significantly inhibit the SGIV-induced cell apoptosis [202]. 

**Figure 3:**
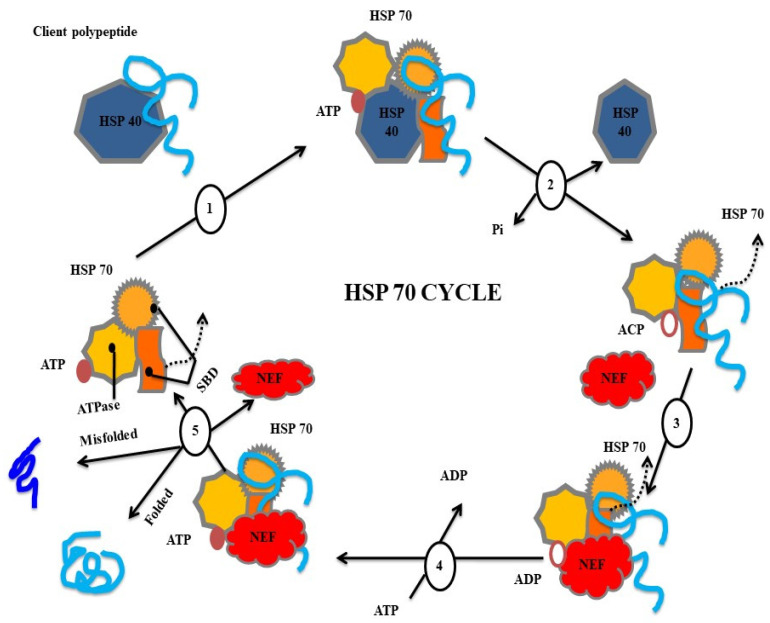
Types of heat shock proteins involved in folding and misfolding mechanisms.

Importantly, abiotic factors also interact with the expression levels of HSPs in aquatic organisms, among which temperature can influence the growth, reproduction, and survival of aquatic organisms (fish and shellfish) and result in serious losses in aquaculture (Figure 3) [203,204]. In a study, the existing HSP20 gene expression was regulated by heat stress [191,192,200,205]. However, few reports provide information about the temperature regulation of HSP20 in fish, and the HSP expression levels in fish under stress factors are poorly understood. Therefore, it is necessary to discuss the findings regarding HSP expression in a range of aquatic organisms with regard to biotic and abiotic stress factors, as the gene expression profile can reveal the importance of their enhancement against foreign stimuli/invaders.

## 5. Conclusions

Our understanding of the chaperone system of HSPs and its significance in farmed aquatic organisms is still limited, but progress is being made in medical and veterinary research. There have been rapid advances in comprehending the fundamental aspects of HSP genes and the effects of their products and their regulation on cell maintenance, as well as cell signaling, inflammation, and the immune response. This knowledge has been applied to various veterinary and human clinical situations, and promising results have been obtained during the initial development of HSP vaccines derived from pathogens. These advancements indicate the potential value of HSPs in numerous areas of aquatic science. Further exploration of the HSP chaperone system and its applications could have significant implications for the health and wellbeing of farmed aquatic animals, providing opportunities for advancements in aquaculture practices, disease prevention, and overall aquatic ecosystem management.

## Figures and Tables

**Table 1:** Expression of HSPs in crustaceans under varying stress conditions and their responses.

Species	Stress Factor	Type of HSP	Protein Response	References
*Tigriopus japonicus*	Environmental toxicants (heat, heavy metals, and endocrine disrupting chemicals (EDCs)	Hsp70	Upregulation	[37,47,68]
	Heavy metal stress	Hsp105/Hsp90/Hsp70	Upregulation	
	Endocrine disruptors	Hsp20	Upregulation	
*Penaeus monodon*	Heat treatment	Hsp90	Upregulation	[48,69]
	pH challenge, osmotic stress, and heavy metal exposure	Hsp60 and Hsp10	Upregulation	
	Salinity stress	Hsp21	Upregulation	
	Oxidative stress: endosulfan and deltamethrin	Hsp90	-	
*Litopenaeus vannamei*	Thermal	Hsp70	Upregulation	[53,70,71]
	Nitrite-N stress	Hsp70	Upregulation	
	Cold shock at 13 °C	Hsp70	Upregulation	
	WSSV infection	LvHSP70	Tenfold upregulation	
*Daphnia magna*	Environmental stresses (cyanobacteria, predation from fish, toxic compounds, temperature)	Hsp60s	Upregulation	[72,73]
	Cadmium and heat stress	Hsp70	Upregulation	
	Environmental	Hsp70	Upregulation	
*Portunus trituberculatus*	Salinity stress	Hsp90, Hsp60	Upregulation	[31]
	Salinity stress	Hsp70	Upregulation	
*Macrobrachium malcolmsonii*	Hg and Cu	Hsp70	Upregulation	[36]
*Macrobrachium rosenbergii*		Hsp70/Hsc70	Upregulation	[74]
*Amphipods*	Cadmium chloride and temperature stresses	Induced by both temperature and toxic stresses	Upregulation	[75]
*Palaemon elegans*	Thermal stress	No significant result		[23]
*Palaemon serratus*				
*Paracyclopina nana*	UV radiation	Hsp60	Upregulation	[34]
*Porcellio scaber*	Metals	Lower hsp70 levels	Downregulation	[76]
*Homarus americanus*	Acute thermal stress, osmotic stress, molting stress	Significant induction of heat shock, hypo-, and hyper-osmotic responses	Upregulation	[77]
*Nephrops norvegicus*				
*Homarus americanus*	Equivalent temperature shift	Hsp70	Upregulation	[77,78]
	Thermal shifts	Hsp90/Hsp70/Hsc70	Upregulation	
*Procambarus clarkii*	Extreme light	Hsp70	Upregulation	[79]
	Help medial giant axons to maintain essential structures and functions	Hsp70	Upregulation	[80]
*Artemia franciscana*	Long-term anoxia	Substantial amounts of p26 translocated into nuclei of anoxic brine shrimp embryos	Upregulation	[81]
	Cd and Zn acute exposure and non-lethal heat shock	Hsp production	Upregulation	[38]
*Artemia sinica*	CO_2_-driven seawater acidification	Upregulated in all treatments	Upregulation	[82]
*Gammarus pulex*	Thermal stress	Hsc70	Upregulation	[83]
	Dissolved humic substances (HSs)	Significantly increased expression of Hsp70	Upregulation	[84]
*Gammarus lacustris* *Eulimnogammarus cyaneus E. verrucosus*	Involved in stress defense system	Hsp70/sHsp	Upregulation	[75]
*Calanus finmarchicus*	Diapause	Hsp70	Upregulation	[85]
*Neohelicegranulatus*	Food	Hsp70	Upregulation	[86]
*Portunus trituberculatus*	Salinity	Hsp70	Upregulation	[31]
*Pachygrapsus marmoratus*	Temperature, salinity, and pH	Hsp70	Upregulation	[23]
Antarctic krills (*Euphausia superba* and *E. crystallorophias*)	Thermal shock	Hsp70	Upregulation	[87]
*E. verrucosus* and *E. cyaneus*	Acute thermal stress	Hsp70	Upregulation	[20]
*Scylla serrata*	Temperature, pathogen, salinity, nitrite stress	Hsp70	Upregulation	[30]
*Niphargus virei* and *N. rhenorhodanensis*	Thermal stress	Hsp70	Upregulation	[88]
*Eriocheir sinensis*	Both low and high salinity	Hsp70	Upregulation	[26]
*Oniscus asellus*	Organic chemicals, metals	Hsp70	Upregulation	[76]
*Metapenaeus ensis*	Exogenous estradiol-17β	Hsp90	Upregulation	[89]
*Marsupenaeus japonicus*		Mjhsp60, Mjhsp70, Mjhsp90	Upregulation	[90]
*Exopalaemon carinicauda*	pH and ammonia-N stresses	Hsp90	Upregulation	[91]
*Eriocheir sinensis*	Glyphosate	Hsp20, Hsp60, Hsp70, HSP90	Upregulation	[92]
	Deltamethrin	Hsp60, Hsp70, Hsp90	Upregulation	

**Table 2:** Expression of HSPs in various parts of fish under stress conditions.

Species	Tissue	Stressor	HSPs	References
*Catla catla*	Larvae	UV-B radiation	Hsp70	[104,105]
	Muscle		Hsp27, Hsp47, Hsp60, Hsp70, Hsp90, Hsp110	
*Channa striata*	Gill, muscle	Heat stress	Hsp27, Hsp47, Hsp60, Hsp70, Hsp78, Hsp90, Hsp110	[106]
*Cirrhinus mrigala*	Liver, gill, brain, kidney	Heat stress	Hsp70	[107,108]
*Danio rerio*	Embryo		Hspb1, Hspb2, Hspb3, Hspb4, Hspb5a, Hspb5b, Hspb6, Hspb7, Hspb8, Hspb9, Hspb11, Hspb12, Hspb15	[109]
*Labeo rohita*	Liver	Arsenic	hsp47, hsp60, hsp70, hsc71, hsp78, hsp90	[107,108,110,111]
	Liver	Starvation/fasting	Hsp70	
	Liver, anterior kidney, spleen	*Aeromonas hydrophila* infection	Hsp30, Hsp70, Hsp90	
*Pethia sophore*	Liver, gill, muscle	Heat stress	Hsp27, Hsp47, Hsp60, Hsp70, Hsp78, Hsp90, Hsp110	[112]
*Rita rita*	Liver, gill	Pollution	Hsp27, Hsp47, Hsp60, Hsp70, Hsp90, Hsp110	[113]
*Salmo salar*	Skeletal muscle	Starvation/fasting	Hsp90α1a, Hsp90α1b, Hsp90α2a, Hsp90α2b, Hsp90ß1a	[114]
*Garra rufa*	Muscle	Naturally living in a hot spring temp. (34.4 °C)	Hsp70, Hsp60, Hsp90, Hsc70, Grp75	[115]
*Squalius torgalensis* and *Squalius carolitertii*	Pectoral, pelvic, upper caudal fins, muscle	20, 25, 30, and 35 °C for 1 °C per day	Hsp70, Hsc70	[116]
*Larimichthys crocea*	Muscle, brain, liver, spleen, kidney, gill, and blood	Low temp. (19 °C) and high temp. (27 and 31 °C)	Hsp27	[117]
*Gadus morhua*	Plasma	Increased temp., 2 °C (2 °C/h) and control 10 °C	Hsp70	[118]
*Fundulus heteroclitus*	Whole organism	Thermal stress from 2 to 34 °C	Hsp70 and Hsp90	[119]
*Carassius auratus*	Cells derived from caudal fin	4 h heat shock form 20 to 40 °C	Hsp30, Hsp70 mRNA	[120,121]
	Brain	2 h heat shock from 22 to 32 °C	Hsp72, hsp90	
*Oncorhynchus mykiss*	Red blood cell	8 h heat shock from 10 to 30 °C	Hsp70 mRNA	[122,123,124]
	Gill, liver, spleen, heart, and head kidney	18 °C were exposed to an elevated temp. (25 °C)	Hsp60 mRNA	
	Liver and heart tissues	8 h heat shock from 13 to 25 °C with 18–24 h recovery	Hsp70, Hsp90	
*Acipense medtrostrs*	Whole larvae	3 day heat shock from 17 to 26 °C at 1.5 °C/h	Hsp72, Hsp78, Hsp89	[125]
*Labeo rohita*	Kidney, gill, liver, and brain	30 day heat shock at 31, 33, and 36 °C	Hsp70	[107]
*Penaeus monodon*	Tail muscle	24 h heat shock from 29 to 35 °C	Hsp70	[126]
*Ictalurus punctuatus*	Muscle	Exposure to low temp. from 25 to 10.5 °C for 14 and 28 days	Hsp70 mRNA	[127,128]
	Tissue	Bacterial infections	Hsp90, hsp60, and shsp families	
*Macrobrachium* *malcolmsonni*	Gill and heart	3 h heat shock from 25 to 32–34 °C and 30 to 36–38 °C with 1 h recovery	Hsp70	[129]
*Macrobrachium* *rosenbergii*	Hepatopancreas and thoracic glands	2 h heat shock form 25 to 30 and 35 °C	Hsp70 mRNA	[74]
*Ostrea conchaphila*	Gill	1 h heat shock from 12–15 to 33–38 °C	Hsp70	[130]
*Ostrea edulis*	Gill	1 h heat shock from 18 to 34 °C with 24 h recovery at 18 °C	Hsp70	[57]
*Channa striata*	Gill	Heat shock treatment at 36 °C for 4/15/30 days	Hsp60, Hsp70, Hsp78	[106]
*Clarias gariepinus*	Embryos	Heavy metals	HSP70	[131]
*Rainbow trout*	Cultured trout cell line	Heat shock and sodium arsenite	Rapid synthesis of trout Hsp70 mRNA	[132]
*Danio rerio*	Brain	37 °C heat stress	Hsp47	[133]
	Embryos	Environmental stress	Hsp70	[134,135,136]
	Early-stage embryos	Heat shock	Hsp90α and Hsp90β genes	
	Embryonic development		Hsp47, Hsp70, and Hsp90	
	Embryonic development		Hsp90 alpha and Hsp90 beta genes	
*Oreochromis niloticus*	Liver, head kidney, spleen, and gill	*Streptococcus agalactiae*	Hs70 family, Hsc70-1, Hsc70-2, and Hsc70-3	[137,138,139]
	Liver, brain, and gill	Cortisol	Hsp70	
	Muscle, gill, and liver	Different degrees of heat (10, 15, 35, 39 °C)	Hsp70	
*Oreochromis niloticus* fingerlings	All organs	Hyperthermal-induced stress	HSP70	[140]
*Garra rufa*	Liver	Elevated water temperature	Hsp70, Hsp60, Hsp90, Hsc70, and Grp75	[115]
*Oreochromis niloticus*		Anoxia stress	Hsp70	[141]
*Sarotherodon melanotheron*	Gills	Environmental salinity	Hsp70	[142]
*Anguilla marmorata*	Liver, intestine, muscle, and heart	*Aeromonas hydrophila* challenge	Amhsp90, Amhsp70	[143]
*Oncorhynchus mykiss*	Gill, liver, spleen, heart, and head kidney	Elevated temperature	Hsp60	[123]
*Oreochromis niloticus*	Gonad, liver, and muscle	Elevated water temperature	Hsp90	[144]
*Miichthys miiuy*	Liver, spleen, and kidney tissue	Bacterial infection	Heat shock protein 90b isoform	[145]
*Boleophthalmus pectinirostris*	Gill, liver tissues	Heat stress conditions	Hsp90AB	[146]
*Dreissena polymorpha* and midge larvae *Chironomus tentans*			Hsp70	[147]
*Fenneropenaeus chinensis*		Microbial pathogens	Hsp70	[148]
		Heat shock and hypoxia	Hsp70	
*Portunus trituberculatus*		Different environmental conditions	Hsp90 genes	[39]
Chinook salmon		Heat shock	Hsp90 genes	[149]
*Cyprinus carpio*	Gill	Ammonia stress	Hsp70	[60]
*Trematomus bernacchii*		Cold shock		[150]
*Pimephales promelas*	Gill, muscle, and brain	28, 31, and 33 °C		[151]
*Palaemonetes pugio*	Muscle	Heat, cadmium, atrazine, and bunker fuel		[152]
*Salmo salar* L.		Anesthesia, formalin exposure, hypoxia, handling, crowding, and cold shock	Hsp70	[153]
*Oncorhynchus kisutch*	Kidney and liver	*Renibacterium salmoninarum*	Hsp70	[154]
Rainbow trout	Anterior kidney	*Vibrio anguillarum*	Hsp70	[155]
*Sparus sarba* Forsskål	Kidney and liver tissue	*Vibrio anguillarum*	Hsp90 and Hsp60	[156]
Brine shrimp/*Vibrio* model		Heat shock at 37 °C *Vibrio campbelli* or *Vibrio proteolyticus*	Hsp70 upregulation	[157]
		Hypothermic shock or acute osmotic	Hsp70 No change	
*Penaeus monodon*		WSSV	Hsp21	[158]
*Vide supra*			Hsp gene downregulation	[156]
Salmonids		*Piscirickettsia salmonis*	Hsp60 and HSP70	[159]
*Oncorhychus mykiss* (Walbaum)		Fish pathogen *Flavobacterium psychrophilum*	Hsp60 and Hsp70	[160]
Brine shrimp		*Vibrio infection*	Hsp70	[157]
*Xiphophorus maculates*		*Escherichia coli*	Hsps	[157]
		Heat-shock-stimulated bacteria	Hsps	[161]

**Table 3:** Mollusk expression of heat shock proteins in different organs with varying stress conditions.

Species	Tissue	Stressor	HSPs	Expression	References
*Corbicula fluminea*	-	High thermal	HSP70, HSP90, and HSP60	Upregulation	[162]
*Mya truncata*	-	Chronic heat shock		Upregulation	[163]
*Codringtonia*	Foot, digestive gland, and genitalia	Short-term heat	HSP70	Upregulation	[164]
*Crassostrea virginica* and *Mercenaria mercenaria*	-	-	HSP60, HSP90, and HSP70	Upregulation	[165]
*Cyclina sinensis*	Hemocytes, hepatopancreas	Cd *Vibrio anguillarum*	HSP70	Upregulation	[166]
*Crassostrea gigas*		Long-term thermal waste	HSP70 and HSP90	Upregulation	[167]
Mid-intertidal limpet *Cellana toreuma*		Thermal conditions	HSP70 and HSP90	Upregulation	[158]
*B. koreanus*		Environmental stressors were reported in copper and UV-exposed	HSP	Upregulation	[168]
*Haliotis tuberculata*		Thermal stress	HSP70	Upregulation	[169]

**Table 4:** Roles of different stressors in the responses and expression of heat shock proteins in insects.

Species	Stress Factor	Type of HSP	Protein Response	References
*Tetraselmis suecica*	Redox- and non-redox-active metals	Small TsHSP20 and large TsHSP70 and 100	Fluctuations	[173]
*Chironomus riparius*	Cadmium	Seven sHSP genes (HSP17, HSP21, HSP22, HSP23, HSP24, HSP27, HSP34)	Downregulation	[174,175]
	Temperature variations	HSP27	Upregulation	
	Cadmium	HSP27	Upregulation	
*Musca domestica*	Thermal and heavy metal	MdomHSP10, MdomHSP27, MdomHSP27.1, MdomHSP27	Downregulation	[176,177,178,179,180]
	Starvation, unsuitable temperatures, bacterial and hazard metal challenge		upregulation	
	Insecticide dimethoate and alkylbenzene sulfonate heat shock, Cd stress, and bacterial challenge	HSP70 and HSP60	Upregulation	
	Development and maturation of eggs	HSP60	upregulation	
	Stress conditions	Small HSPs	Upregulation	
*Drosophila melanogaster*	Expressed highly in gonads and nervous system	HSP23, HSP26, and HSP27	Upregulation	[181]
*Sarcophaga crassipalpis*	Cold-induced diapause	HSP23	Upregulation	[182]
*Plutella xylostella*	Heavy metals	sHSPs	Upregulation	[183]
*Galleria mellonella*	*Conidiobolus coronatus*-induced infection	HSP90, HSP70, HSP60, HSP27	Upregulation	[184]

## Data Availability

The data in this study will be disclosed upon request.
